# Psychological Factors, Leisure Activities, and Satisfaction during the COVID-19 Pandemic: A Cross-Sectional Study in Eleven Spanish-Speaking Countries

**DOI:** 10.3390/ijerph182111104

**Published:** 2021-10-22

**Authors:** Paula Hidalgo-Andrade, Clara Paz, Carlos Hermosa-Bosano, Javier García-Manglano, Charo Sádaba-Chalezquer, Claudia López-Madrigal, Cecilia Serrano, Aurelio Fernández-Zapico

**Affiliations:** 1 Escuela de Psicología, Universidad de Las Américas, Quito 170517, Ecuador; paula.hidalgo@udla.edu.ec (P.H.-A.); carlos.hermosa@udla.edu.ec (C.H.-B.); 2 Instituto Cultura y Sociedad, Universidad de Navarra, 31008 Pamplona, Spain; jgmanglano@unav.es (J.G.-M.); aefernandez@unav.es (A.F.-Z.); 3 Facultad de Comunicación, Universidad de Navarra, 31008 Pamplona, Spain; csadaba@unav.es; 4 Facultad de Educación y Psicología, Universidad de Navarra, 31008 Pamplona, Spain; clopez.30@unav.es; 5 Departamento de Sociología, Universidad Católica de Milán, 20123 Milán, Italy; anacecilia.serranonunez@unicatt.it

**Keywords:** lifestyle, COVID-19, satisfaction, leisure, self-esteem, self-control, emotional stability, psychological factors

## Abstract

The COVID-19 pandemic has affected the lives of millions of people worldwide. This study aimed to analyze the effects of several psychological factors (self-esteem, self-control, and emotional stability) over lifestyle-related variables (time spent on leisure activities) and the levels of satisfaction (family, friends, work, and leisure satisfaction) experienced during the COVID-19 outbreak. Data for this article were retrieved as part of a cross-sectional international study conducted in eleven Spanish-speaking countries between March and September 2020. The analyses were conducted using the responses of 9500 persons (65.95% women, 34.05% men). Structural equation modeling was used to test the direct and indirect effects of the psychological variables on satisfaction variables mediated by the time engaged in leisure activities. Our model indicated that psychological factors significantly predicted the amount of time spent in leisure activities and satisfaction. Overall, results indicate that self-esteem is a relevant psychological factor to consider in the development of psychological interventions directed at promoting healthy lifestyles. Nevertheless, further research is needed to validate the direction of the associations found in this study.

## 1. Introduction

The COVID-19 pandemic, a disease caused by the SARS-CoV-2 virus, has affected the lives of millions of people. By September 2021, more than 222 million people had been infected with the virus and there had been an estimated number of 4.5 million deaths across the Globe. Fortunately, the administration of vaccines has increased significantly, allowing many people to resume their day-to-day activities. At the time when this manuscript was written, there had been 5.3 billion doses administered worldwide [1].

Although many people with COVID-19 are asymptomatic and do not develop severe complications, the rapid spread of this disease in the first months of 2020 created excessive burdens in the health systems of many countries and urged governments to implement different preventive measures including lockdowns, curfews, teleworking, online education, physical distancing, among others. Research has demonstrated that these measures had a toll in people’s mental health and psychological well-being. However, there is still missing information regarding the lifestyle changes that occurred during the pandemic and the series of psychological variables associated with these changes.

### 1.1. Leisure Activities and Lifestyle Changes during COVID-19

The severity and novelty of some of the sanitary control restrictions to control the pandemic had a direct effect on how people conducted their lives. Most people had to adopt teleworking to remain productive and ensure stable incomes, some adopted child-care responsibilities—including homeschooling—and many others engaged in a series of household duties that they had never done before. The pandemic also implied changes in the labor market [2,3]. Research from the International Labor Organization suggests that during the second quarter of 2020, 560 million people were working from home and 144 million people worldwide lost their jobs [4].

These shifts in people’s lives may have altered their normal day-to-day endeavors, including the time spent doing leisure activities such as exercising, using social media, listening to music, watching television, and playing video games. As McDowell et al. [2] suggest, for some individuals, staying at home may have increased their opportunities to engage in leisure activities due to their reduced commuting time and their increased dependency on technological devices.

Research related to the effects of COVID-19 on people’s lifestyle is still in its initial stages. In a study conducted by McDowell et al. [2] using a sample of 2303 US adults, the authors found that participants whose employment status changed due to COVID-19 (i.e., those who worked from home, or lost their job), reported higher sedentary behavior and screen time. However, they did not find differences in the levels of physical activity. In another study with 3052 US participants, Meyer et al. [5] found a reduction in the weekly physical activity levels among those who reported being physically active prior to the pandemic, whereas it remained unchanged for those who were not physically active. In addition, the authors reported correlations between decreased physical activity, higher screen time, and higher depressive symptoms, loneliness, stress, and lower positive mental health. Research in other countries has shown similar results. In a study conducted by Salman et al. [6] in Kuwait, results indicated that around 34% of the sample exercised more irregularly than before the COVID-19 outbreak and only half (48.8%) reported exercising for at least 30 minutes three or more days a week. Similarly, in a study in Qatar carried out by Hermassi et al. [7] with 1114 people, results showed an increase of sedentary behavior and decreased physical activity during the COVID-19 pandemic.

Other studies have analyzed the effects of the pandemic in other leisure behaviors including the use of social media and technological devices. In general, research indicates an increase in the amount of self-reported screen time using technological devices [8,9]. At the beginning of the COVID-19 pandemic, digital devices were used for entertainment purposes, such as watching television, movies, and series through streaming services (e.g., Netflix, Amazon Prime, HBO). Other common motivators for digital use included connecting with others and socializing through social media platforms (e.g., Facebook), as well as shopping for groceries and other goods. Although many people may have experienced a sense of relief owing to the use of technology, research indicates that increased social media usage and technology consumption relates with poorer mental health [9]. In a comparative study using samples from Norway, UK, US, and Australia, Geirdal et al. [10] found that highly frequent social media users were more inclined to have poorer mental health, quality of life, and well-being, as well as higher feelings of loneliness. However, much more research needs to be completed to clarify the causal directions between these variables. For example, these authors suggest that people with higher levels of mental health may seek social media for more positive purposes, including distraction, recreation, and relaxation.

### 1.2. Psychological Factors and Leisure Activities during the Pandemic

Psychological variables may influence the type of leisure activities people engage in and the levels of satisfaction associated with them. These factors include self-control, self-esteem, and emotional stability, and they, in turn, might be affected by sociodemographic variables such as sex and age. However, more research directed at analyzing how psychological factors relate to the levels of satisfaction—including leisure-related satisfaction—is needed.

#### 1.2.1. Self-Esteem

Self-esteem can be defined as a person’s evaluative judgment of their self [11]. Previous research has found that self-esteem impacts a series of aspects including interpersonal relationships, decision-making processes, mental health indicators, and overall well-being [12]. Stressors during the COVID-19 pandemic, such as the experience of uncertainty, social distancing, confinement, inadequate information, loneliness, lack of outdoor activities, financial loss, and obstacles to obtain food and water may have restricted the satisfaction of basic needs which resulted, for some individuals, in the deterioration of their self-esteem and hence, their well-being [13].

In this scenario, the adoption of leisure activities as part of people’s daily routines has been promoted by several organizations as a way to cope with the stress that has resulted from the pandemic [14,15]. Previous research indicates that engagement and satisfaction with these activities are positively related with high levels of self-esteem at different stages of the life span [16,17,18]. Moreover, self-esteem has been positively associated with leisure activities that include physical activity [19]. Interestingly, with the onset of the pandemic, many people experienced an increase in the amount of time engaged in technologically driven, screen-based activities, which in turn seems to be negatively related to self-esteem [20]. Thus, there are still scarce data regarding the impact of self-esteem on the amount of time spent conducting these activities. To the best of our knowledge, sex and age differences have also not been reported.

#### 1.2.2. Self-Control

Self-control refers to a person’s capacity to change and adapt the self to the conditions and restrictions imposed by the outside world. It has been conceptualized as the ability to ignore or change one’s inner responses, interrupt undesired behavioral tendencies, and desist from acting on them [21]. Previous studies have suggested that self-control is associated with higher levels of well-being [22] and lower levels of anxiety and depression among those with higher self-control [22,23]. Recent studies have proposed self-control as an important psychological resource associated with the regulation of negative mental health effects of the COVID-19 pandemic [24,25]. Individuals with higher levels of self-control may use more positive coping strategies and restrain from using negative ones to face the pandemic [25]. Moreover, individuals with higher self-control may also be more likely to design and act upon plans to face the pandemic, regulate their emotions, and adhere to the government’s prevention protocols and guidelines (e.g., social distancing, confinement) to fight against COVID-19 [25].

Regarding leisure activities, studies conducted before the pandemic show that self-control has been associated with higher leisure time, physical activity, aerobic fitness, muscle fitness, and lower body mass index [26]. In another study, leisure time physical activity, goal progress, and self-efficacy partially mediated the relationship between self-control and subjective well-being [27].

To the best of our knowledge, there are no studies regarding the relationships between physical activity and self-control during the pandemic. Regarding social media use, studies have found that media users often have difficulties regulating their behaviors [28]. In a study conducted by Hofmann et al. [29], the authors found that the desire to engage in social media is related to self-control failures. This effect has been described regarding social media and other leisure-related activities, including watching television [30].

In relation to sex and age differences, studies conducted before the pandemic have shown that men and women tend to have similar capabilities for self-control. However, men may have higher difficulties to regulate antisocial or problematic impulses that may lead to higher drug and alcohol abuse, and stronger sexual and aggressive impulses [22]. Similarly, studies have found that younger adults may have weaker self-control, leading to problematic behavior [22]. The possible effects of sex and age in the relationship between self-control and leisure behaviors are not clear.

#### 1.2.3. Emotional Stability

Emotional stability refers to a person’s ability to remain stable and balanced in opposition to the tendency of easily experiencing negative emotions [31]. Studies conducted before the pandemic point out that emotional stability predicts the amount of time spent on and the type of chosen leisure activities [32]. In a meta-analysis of 16 national surveys conducted by Sutin et al. [32], the authors found that participants with higher levels of emotional stability pursued more physical activities than those with lower levels; this difference was stronger among younger adults, and nonsignificant effects were found based on sex. Higher levels of emotional stability have also been associated with less time engaging in sedentary leisure behaviors, such as watching TV; age and sex, however, do not seem to moderate this association [33]. Regarding internet use and social media, people with higher levels of emotional stability, especially those who are older, spend less time on the internet and show lower cellphone use [34,35,36].

In addition, studies indicate that higher levels of emotional stability are positively linked with overall life satisfaction [37,38]. Studies conducted during the pandemic have also indicated that high levels of emotional stability are positively correlated with well-being [39,40,41].

### 1.3. Satisfaction with Interpersonal Relations, Work, and Leisure during COVID-19

Life satisfaction is a commonly studied component of psychological well-being. It is often regarded as a person’s subjective assessment of their overall life [42] as well as “the extent to which (he/she) finds life rich, meaningful, full, or of high quality” [43]. Life satisfaction can be studied by taking a global approach (i.e., overall life satisfaction) or by analyzing more specific domains including the quality of relationships with friends and family, and other life areas, such work and leisure.

Psychological research has found that life satisfaction is related to the degree a person feels hope and meaning in life [44,45]. A study conducted during the pandemic in Qatar showed a decrease in life satisfaction by 55% in males and 57% in females [7]. In another study, life satisfaction was found to have a mediating effect on the relationship between hope and anxiety, and the relationship between hope and COVID-related stress [44,45]. Personality traits such as extraversion and neuroticism may influence the subjective experiences of life satisfaction [46]. Being male, being employed, having had less time of confinement, having had higher access to more information, and having had private access to an outside space were also associated with higher levels of life satisfaction [47].

The pandemic brought changes in people’s lifestyles and the amount of time and types of leisure activities they engaged in. Thus, leisure satisfaction becomes a relevant variable to deepen psychologists’ understanding of the effects of the pandemic, especially since it has been previously related to happiness [48] and subjective well-being [49]. Leisure satisfaction can be understood as “the degree to which people have experiences in their lives that fulfill needs or desires for expression, rest and relaxation, entertainment, and other personal interests” (p. 1) [50]. Research analyzing this variable during the pandemic found that leisure satisfaction predicted the levels of job motivation of healthcare workers in Turkey [51]. Much more research is needed to better unpack the role that leisure activities play in life satisfaction and well-being.

### 1.4. The Current Study

Most psychological studies on COVID-19 have focused on the negative psychosocial consequences of social and mobility restrictions in mental health outcomes such as anxiety and depression. However, given the different measures adopted around the globe to stop the spread of the virus, it is important to look at other aspects of human behavior such as the amount of time spent conducting leisure activities and domain-specific levels of satisfaction. Thus, this study aimed to describe the effects of psychological variables (i.e., self-esteem, self-control, and emotional stability) on the time spent on specific leisure activities (i.e., sports, hobbies, social networks, and watching TV shows) and the levels of satisfaction (i.e., friends, family, work, and leisure) during the COVID-19 pandemic of people from 11 Spanish-speaking countries. We will test the direct effects of the psychological variables on satisfaction measures and the indirect effects of the time spent in leisure activities.

We hypothesize that higher levels of self-esteem, self-control, and emotional stability will be positively related to the amount of time engaged in nonscreen leisure activities, such as playing sports or other hobbies; in contrast, psychological variables will be negatively related to leisure activities that involve sedentary behaviors such as the use of social media and watching series, TV, movies, etc. Additionally, time spent in leisure activities will mediate the relationship between psychological factors and satisfaction with personal relationships, job activities, and leisure time.

## 2. Materials and Methods

### 2.1. Participants

This study followed a cross-sectional design. To take part of the study, participants had to be Spanish-speaking adults—older than 18 years—who voluntarily wanted to take an online survey. Participants were excluded if they did not meet these requirements. Those participants who voluntarily abandoned or did not complete the survey were eliminated during the analytic phase. None of the participants received monetary compensation for taking the survey.

### 2.2. Instruments

The online survey assessed and analyzed the constructs that follow:

#### 2.2.1. Self-Esteem

We used the Spanish version of the Rosenberg self-esteem scale [52,53]. It consists of 10 items such as “You feel as valuable as other people” and “You are satisfied with yourself” with a four-point answer scale ranging from “totally agree” to “totally disagree” A total score between 10 and 40 points was obtained by adding the responses to each item. The cutoff points to establish self-esteem levels follow: low (0–25), moderate (26–29), and high (20–40). Reliability analyses in this sample indicated adequate coefficients (α = 0.87).

#### 2.2.2. Self-Control

We selected an item from the Spanish version of the self-control scale [54,55]. Respondents were asked “How often do you find it difficult to control your impulses?” using a four-point answer scale ranging from 1 “often” to 4 “never”. Psychometric analyses in the sample of this study yielded acceptable reliability levels (α = 0.84).

#### 2.2.3. Emotional Stability

We used the Spanish version of the ten item personality inventory [56,57]. This scale is designed to obtain the big five personality traits: extraversion, openness, neuroticism, agreeableness, and conscientiousness. It consists of a 10-item scale with a four-point answer scale ranging from 1 “not at all” to 4 “a lot”. Reliability and validity values were adequate (α = 0.73). In this article, we focused on neuroticism. Respondents had to indicate if they see themselves as “calm, emotionally stable” or “anxious, easily upset”. This factor evaluates the degree of emotional stability.

#### 2.2.4. Leisure Activities

We assessed the amount of time people spent on leisure activities through the following self-reported questions: “Approximately, how many hours a week would you say you exercise or practice a sport?”, “How many hours a week do you spend on hobbies that do not require a smartphone or a screen?”, “How many hours a day do you use devices to watch series, videos, movies, etc.?”, and “How many hours a day do you use devices for social networks (Instagram, TikTok, etc.)?”. In this study we report the responses in weekly hours.

#### 2.2.5. Satisfaction

Participants indicated their level of satisfaction in four different areas: their relationship with their family, their relationship with friends, and their work and leisure time. Participants were asked the following question: “Indicate how satisfied you feel with these aspects of your life?” A four-point Likert-type scale was used to record the answers; responses ranged from “unsatisfied” to “very satisfied”.

#### 2.2.6. COVID-19 Restrictions

We used an index of stringency built by the Oxford COVID-19 Government Response Tracker (OxCGRT). For each respondent, based on their country and date of response, it assigns a number from 0 to 100 according to a “composite measure based on nine response indicators including school closures, workplace closures, and travel bans, rescaled to a value from 0 to 100 (100 = strictest). If policies vary at the subnational level, the index is shown as the response level of the strictest subregion.” [58].

### 2.3. Procedures

We conducted a cross-sectional study, which was part of a broader research project of the “Youth in Transition” research group at the University of Navarra. The sample analyzed in this article was taken from the broader study. A questionnaire was developed by the researchers using validated scales and self-reported questions. This questionnaire was then distributed through an online platform (Google Forms). Because of the questionnaire’s format, the sample was limited to those with internet access. The participation of this questionnaire was voluntary and anonymous. To take part in the study, participants had to agree with the specifications provided in the informed consent page presented at the beginning of the survey. Data were collected from March 30 to September 30, 2020, in eleven Spanish-speaking countries where COVID-19 sanitary measures were implemented. A team of collaborators in each participating country were responsible for the distribution of the questionnaire (see Acknowledgments). The research project was approved by the Ethics Committee of the University of Navarra (Ref. 2020.087).

### 2.4. Data Analysis

**Figure 1:**
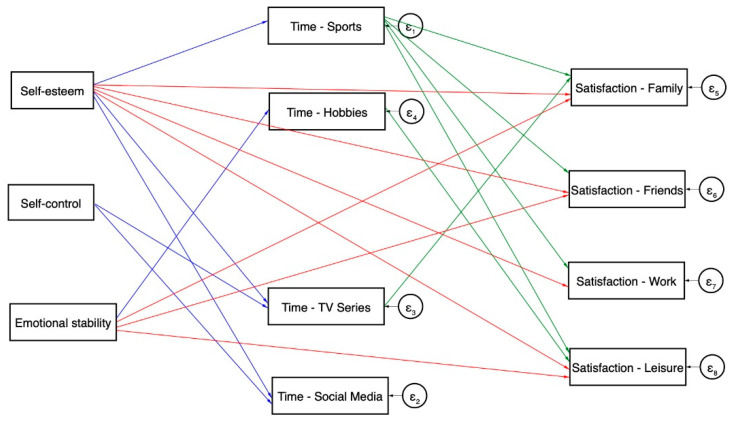
Direct effects tested in the structural equation model. Note: Blue arrows indicate the direct effects of psychological variables on time spent in different activities, red arrows indicate the direct effects of psychological variables on satisfaction on each area, and green arrows indicate the direct effect of the time spent for each leisure activity on the satisfaction on each area. Control variables (age, sex, and stringency) affecting activities and satisfaction are not shown.

To test our hypotheses, we performed descriptive, correlational, and multivariate analyses using structural equation models (SEM). First, we explored descriptively the variables and the relationships between them. Then, we carried out a structural equation analysis (Figure 1), to better understand the relationship between variables. The psychological variables (self-esteem, self-control, and emotional stability) were considered as independent variables. Leisure activities (time spent on hobbies, sports, social media, and TV series and video watching) were used as mediators, and satisfaction (family, friends, work, and leisure) were considered as the dependent variables. Age, sex, and stringency (COVID-19 restrictions) were used as control variables in the analysis. SEM allowed us to test the direct and indirect effects between the variables, and the prediction effects that the mediating variables have in the model. Comparative fit index (CFI) and root mean square error of approximation (RMSEA) were used to interpret the fit of the structural equation model (SEM). Analyses were conducted using STATA (version 16, StataCorp, College Station, TX, USA).

## 3. Results

A total of 9500 Spanish-speaking individuals were recruited for this study; 65.95% were women and 34.05% were men. Participants were from 11 different Hispanic countries. As seen in Table 1, the country with the most participants was Guatemala (*n* = 1619) representing 17% of the sample, and Chile was the country with the least number of participants (*n* = 504), representing only 5.3% of the sample. The age of participants ranged from 18 and 80 years old with a mean of 35.2 (*SD* = 14.57) and were distributed as follows: 18–22 (27.49%), 23–29 (18.26%), 30–39 (16.92%), 40–49 (18.52%), 50–59 (12.25%), and 60+ (6.56%). Regarding socioeconomic status, participants were asked “In what socioeconomic status would you place yourself and your family?” and 40.86% of our sample identified their family as high, 47.73% as middle, and 11.41% as low. More than half (62.51%) of our sample reported having completed higher education studies.

Descriptive data on leisure activities (see Table 2) indicated that time spent in online activities was higher than nonscreen activities. Specifically, the use of social media was the activity in which people spent more hours per week, followed by watching TV series and videos. Regarding sex, men spent more time engaged in nonscreen activities (sports and hobbies) than women. On the contrary, women spent more time in online activities, especially social media. In relation with age, time spent in online activities decreased as people got older. On the contrary, nonscreen activities seemed to increase slightly with age.

Psychological variables were also analyzed descriptively using the weekly hours spent on leisure activities. Results revealed that high levels of self-esteem, self-control, and emotional stability were related with more time spent in sports and hobbies, whilst low levels of these psychological variables related to more hours using social media and watching TV series and videos.

### 3.1. Bivariate Correlations

Positive and negative correlations were found between psychological factors (self-esteem, self-control, and emotional stability), leisure activities (time spent on sports, hobbies, social media, and TV series/video watching), and satisfaction with family, friends, leisure, and work activities (see Table 3). All the psychological factors had a significant negative correlation with social media time and TV series/video watching. Additionally, time spent on hobbies correlated negatively with social media use.

Both online leisure activities, time spent on social media and TV series/video watching, correlated negatively with the four domains of satisfaction (family, friends, leisure, and work activities).

### 3.2. Structural Equation Model

**Figure 2:**
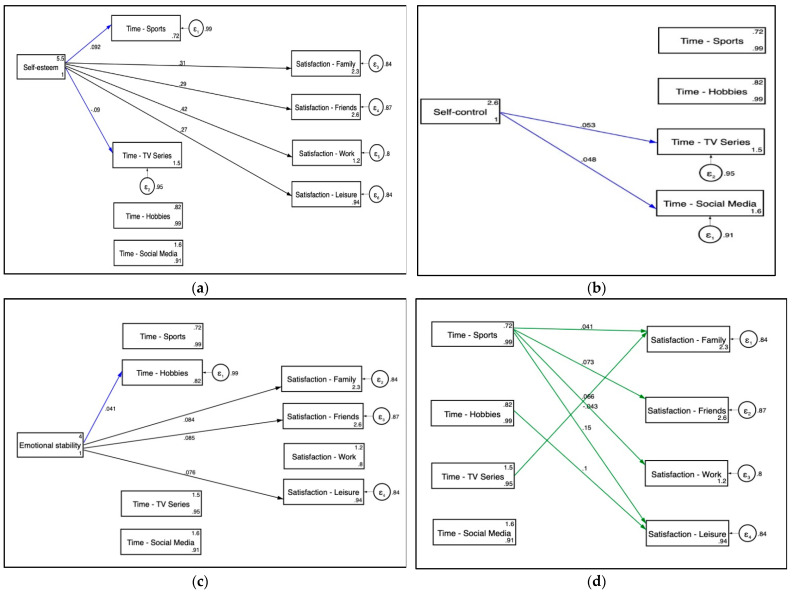
Significant paths on time spent on leisure activities and satisfaction. (**a**) Self-esteem on leisure activities and satisfaction. (**b**) Self-control with time spent on leisure activities. (**c**) Emotional stability on leisure activities and satisfaction. (**d**) Time spent on leisure activities with satisfaction.

In order to test the effects of psychological factors on satisfaction, mediated by leisure activities, we fitted a structural equation model. The first model indicated a good fit with a comparative fit index CFI = 0.99, a Tucker–Lewis fit index (TLI) = 0.97, and the RMSEA = 0.02. Due to the clustered nature of our data, standard errors had to be controlled by groups. If data were considered individually, the standard error will diminish artificially, increasing the probability of type I error and turning significant some of the relationships. Hence, we ran the same model with clustered standard errors by country with a SRMR of 0.005. This final model (see Figure 1) revealed that psychological factors have a significant predictive effect both on leisure activities and satisfaction. However, the explicative power of it is relatively low because the explained variance of leisure activities is between 1 and 9%, and between 13 and 20% regarding satisfaction. A more detailed overview of the significant paths is shown in Figure 2.

### 3.3. Effects of Psychological Factors on Leisure Activities

The direct linear effects revealed predictive relationships between the assessed psychological factors and leisure variables (see Table 4). Self-esteem had greater potential and significance for positively determining leisure time spent in sports and hobbies, and negatively determining social media use and TV series and videos. Self-control negatively influenced time for sports, social media, and TV series and videos. As for emotional stability, a positive effect on time for hobbies and a negative effect for social media use were found. Being a woman (sex) was negatively related to sports time and positively related to social media use, while age had a negative association with sports, social media, and series.

### 3.4. Total and Mediated Effects of Psychological Factors and Leisure Activities on Satisfaction

As shown in Figure 1, we hypothesized that satisfaction with family, friends, work, and leisure would be directly affected by self-esteem, emotional stability, time spent in sports, hobbies, social media, and watching videos, controlling by age, sex, and COVID-19 restrictions. In addition, we expected that the effect of self-esteem, emotional stability, and self-control would also be mediated by leisure activities. That is, that the total effect would be the sum of the direct and the indirect effects, except for self-control, which would only affect satisfaction through the time spent on certain activities. Family satisfaction was positively associated with leisure time in sports, self-esteem, and emotional stability, and negatively determined by time spent on TV series and watching videos. Similarly, satisfaction with friends was also strongly positively influenced by leisure time in sports, self-esteem, and emotional stability (see Figure 1). Work satisfaction, however, was not related with emotional stability. Results suggest that it was determined by leisure time in sports and self-esteem; time spent on TV series and videos had a slight but significant effect as well. Lastly, as seen in Table 5, leisure satisfaction was positively associated with self-esteem, emotional stability, sports, and hobbies, and negatively affected by self-control. The indirect linear effects are the effects the independent variables had over the satisfaction variables, mediated through the four leisure activities: time spent on sports, hobbies, watching series/videos, and social media use. Regarding family satisfaction, the total effect of self-esteem was *β* = 0.323 and *p* < 0.001, while part of this effect (*β* = 0.008, *p* < 0.001) passed through sports and watching series and videos. That is, self-esteem affected time in sports and series, as shown in Table 4, and these variables also affect family satisfaction. On the contrary, the effect of emotional stability did not seem to be mediated by leisure activities. A similar result occurred for satisfaction with friends. While part of the effect of self-esteem was mediated by the time spent with sports, emotional stability did not show a significant indirect effect. As for work satisfaction, the effect of self-esteem appeared to be slightly mediated by sports and series and videos. Lastly, the effect of self-esteem on leisure satisfaction was significantly mediated by sports and hobbies while the effect of emotional stability was only mediated by hobbies. Furthermore, the total effect of self-control on leisure satisfaction appeared to be mediated by time spent in sports, which was the only variable affected by self-control and, at the same time, affected leisure satisfaction.

## 4. Discussion

This study aimed to analyze the effects of several psychological factors (self-esteem, self-control, and emotional stability) over lifestyle-related variables (time spent on leisure activities) and the levels of satisfaction (family, friends, work and leisure satisfaction) during the COVID-19 pandemic. We used a large sample of 9500 individuals from 11 Spanish-speaking countries. A structural prediction analysis (SEM) was performed to test the direct and indirect effects between the mentioned variables. Our model indicated that psychological factors significantly predicted the amount of time people spent in leisure activities as well as their levels of satisfaction. The results of the study confirmed our hypothesis that psychological factors are related to leisure activities and to satisfaction in the interpersonal, occupational, and leisure domains.

### 4.1. Psychological Factors on Leisure Activities

Psychological factors explained between 1 and 9% of the variance of the model. Regarding self-esteem, we found that it positively predicted the amount of time people spent on leisure activities such as playing sports and other hobbies, as seen in prior studies [19], while it negatively predicted the amount of time spent on leisure activities such as social media use and watching TV series and videos. These results probably suggest that people with high levels of self-esteem preferred to spend their time doing activities that do not require the use of a screen. It is possible that they were more creative trying to find ways to develop those activities within their home, despite the restrictions of the pandemic.

The model also showed that self-control significantly and negatively predicted the time spent on activities such as watching TV series and videos and checking social media apps. These results are consistent with other studies conducted before the pandemic [28,30], indicating that persons with high levels of self-control spend less time in front of a screen than those with low levels of self-control. Moreover, the evidence suggests that people with high levels of self-control are more prone to spend time doing physical activity [26]. In the present study, the model indicated a negative relationship between self-control and time playing sports. A possible explanation for this finding is that people with high levels of self-control were more likely to adhere to the government’s guidelines to fight against COVID-19, as indicated by Lie and colleagues [25], thereby avoiding sports that probably required them to transgress indications of social distancing and confinement.

Finally, we found that emotional stability significantly predicted the amount of time spent using social media; this relationship was negative, as found in previous studies [34,35,36]. It is possible that these results highlight the fact that people with high levels of emotional stability are better able to regulate the amount of time spent using social media and probably spend time engaging in other forms of leisure activities. The fact that we found a positive predictive association between emotional stability and time spent on hobbies possibly supports this interpretation, since hobbies are not necessarily carried out using technological devices. Surprisingly, we did not find evidence that emotional stability predicted the amount of time spent playing sports, even though we expected a significant correlation, as had been widely demonstrated in other studies conducted before the pandemic [32,59]. This difference might be explained by the fact that most of the previous studies analyzed physical activity, a wider construct that goes beyond sports engagement. We believe playing sports is a much more specific activity and may require open spaces and contact with other persons. In the context of the pandemic, this activity was limited as most of the people who answered the survey were in confinement or had restrictions for physical contact, thereby explaining the weakness of this relationship.

### 4.2. Leisure Time on Satisfaction

We also found that lifestyles measured in this study—as the amount of time engaged in leisure activities—had a direct effect on satisfaction levels. Out of the four different activities explored in this article, only time spent on social media did not have effects on any of the satisfaction variables. This shows that social media does not necessarily contribute or diminish the perceived satisfaction in any of the explored domains.

In contrast, the time spent doing sports, hobbies, and watching series and videos significantly predicted higher levels of leisure satisfaction. This effect could be explained by the fact that leisure satisfaction involves different experiences that involved varied personal interests [50]. Thus, investing time in any kind of activity will increase this type of satisfaction.

Regarding the levels of satisfaction with work and family, we found that these variables were positively predicted by time spent on sports and negatively determined by the amount of time watching series and videos. This could indicate that there is a difference between the activities that could include group dynamics or conversations (sports) and those that are enjoyed individually (series and videos). In addition, activities such as watching series and videos may negatively affect work and family satisfaction possibly because they are more time-consuming than sports and imply higher distractions, especially with current platforms that grant access to hundreds of options that people can watch in one sitting. In fact, data from one of those platforms, Netflix, show that binge-watching is common among its users [60]. Previous research in Southeast Asian countries showed that people who binge-watched during the pandemic experienced sleep disturbances, missed work, and had conflicts with others due to that behavior [61].

Regarding satisfaction with friends, results indicated that it was positively predicted by time spent on sports. This result shows that sports could involve positive feelings and a sense of belonging because many sports are group activities that remained during the pandemic, even virtually [62]. A study shows that people who participated in organized sports before the pandemic and who continued to participate in structured programs during lockdown showed more resiliency and reported higher health and satisfaction with life [63].

Confinement only negatively predicted satisfaction with friends. As previous research suggests, contact with friends and the aspiration for contact reduces loneliness, thus increasing well-being [64]. This result highlights that the pandemic reduced the time one could spend with people outside our close family or social circle, especially at the beginning of the pandemic when social interactions were limited to avoid the spread of the virus.

### 4.3. Psychological Factors on Satisfaction

Results indicate that self-esteem and emotional stability had direct positive effects on participants’ levels of satisfaction with their family and friends. Previous research found that self-esteem is related to positive interpersonal relationships, thus increasing the likelihood of experiencing high-quality, fulfilling relationships with significant others [65]. People with high levels of emotional stability may also experience more satisfying relationships because of their low levels of conflict, higher calmness, and tranquility [66].

Regarding work satisfaction, we found that self-esteem was the only variable that positively predicted this outcome. This result is consistent with research indicating that individuals with high self-esteem tend to be optimistic in the face of adversities [67]. It is likely that people with higher levels of this variable view the pandemic as a temporary situation, approaching work in a more optimistic and positive way. It is also possible that people with higher self-esteem choose occupations that are consistent with their interests [67], making it more likely for them to experience satisfaction, even amid a health crisis such as the pandemic.

In addition, we found that all the psychological variables evaluated (i.e., emotional stability, self-esteem, and self-control) predicted levels of satisfaction with leisure time. High levels of self-control may be related to higher levels of leisure satisfaction because it allows people to effectively divide their time into several tasks, including their own personal time. This may be particularly relevant amid the COVID-19 pandemic, in which many people struggled to keep time for themselves.

It should be noted that all these relationships were weak. Previous research has indicated that the associations between personality characteristics and variables such as life satisfaction consist of an intricate network of direct and indirect pathways [66]. In these series of relationships, variables such as an individual’s life circumstances, health status, physical fitness, and the presence of diseases come into play [66]. Thus, it is plausible that the observed weak effects were explained in part due to the number of nonmeasured predictors that contribute to people’s perceptions of satisfaction in their different life domains.

### 4.4. Indirect Effects through Time Spent on Leisure Activities

In this study, we tested the indirect effects of psychological factors on satisfaction through time spent on leisure activities. As far as we are concerned, this is the first time these paths were evaluated, and they help to provide some recommendations. Overall, our results indicate that self-esteem seems to be the most relevant psychological factor when explaining satisfaction. Emotional stability and self-control only predicted satisfaction with leisure activities, which is specific to the mediators tested. Thus, we believe self-esteem could be a target for future interventions, since it will promote better use of leisure time and that will result in higher levels of satisfaction. One useful intervention could be the use of mindfulness since there is evidence indicating that its constant practice results in increasing self-esteem [68].

### 4.5. Limitations

This study has several limitations that must be acknowledged. First, data collection was conducted using an online survey, thus limiting the characteristics of the population who had access to it. Second, while we believe the analysis of mediation effects between the different variables strengthens our study, the data we used were collected only at one assessment point, thus limiting the potential causal conclusions that might be drawn from this study. We consider our analyses to be exploratory in nature, thus we recommend future research to include data collection at different assessment points to test our results and identify whether the tested pathways are correctly interpreted. Another limitation is that the variable self-control was measured using just one question; we believe future research could use more appropriate scales to evaluate that specific construct.

## 5. Conclusions

Despite its limitations, the large number of participants from 11 Spanish-speaking countries in this study provides a snapshot on how psychological factors affected the time spent in leisure activities and how that mediates satisfaction in four areas (family, friends, work, and leisure) during the COVID-19 pandemic. Our results suggest that self-esteem is a relevant psychological factor that plays a role in the achievement of satisfaction. It is possible that future initiatives could try to intervene in the development of self-esteem. Mindfulness and other techniques could be useful for these purposes. However, given that this trait might be difficult to target directly, we believe future interventions could also promote modifiable practices such as the amount of time people spend engaged in sports and other nonscreen-dependent hobbies. As Salman et al. [6] suggest, increasing the time in sports and physical activity could also have a positive effect in promoting other healthy behaviors such as better sleeping, healthy eating, and the reduction of psychological distress. Finally, we believe future research is needed to further understand how these variables interact in pursuit of healthy lifestyles and well-being during stressful times.

## Figures and Tables

**Table 1:** Number of participants, mean age, and sex distribution by country.

Country		Age	Sex	
	*N* (%)	*M* (*SD*)	Women (%)	Men (%)
Argentina	547 (5.76)	38.44 (15.33)	73.13	26.87
Chile	504 (5.31)	37.37 (14.45)	69.64	30.36
Colombia	770 (8.11)	38.36 (14.70)	75.19	24.81
Ecuador	766 (8.06)	32.36 (12.54)	64.23	35.77
El Salvador	641 (6.75)	30.35 (13.15)	60.53	39.47
Guatemala	1619 (17.04)	34.69 (14.32)	63.19	36.81
Mexico	1084 (11.41)	36.42 (14.43)	64.94	35.06
Peru	1083 (11.40)	29.16 (11.96)	64.54	35.46
Spain	986 (10.38)	37.28 (14.03)	61.76	38.24
Uruguay	876 (9.22)	36.72 (16.21)	68.26	31.74
Venezuela	624 (6.57)	39.10 (15.77)	67.63	32.37
Total	9500 (100)	35.18 (14.57)	65.95	34.05

**Table 2:** Average hours per week of sports, hobbies, social media, and series by sex, age, and different levels of self-esteem, self-control, and emotional stability.

	Sports	Hobbies	Social Media	Series/Videos
	*M* (*SD*)	*M* (*SD*)	*M* (*SD*)	*M* (*SD*)
Total	3.24 (3.16)	4.72 (5.52)	21.07 (22.06)	16.78 (16.26)
Sex				
Men	3.48 (3.32)	4.85 (5.69)	19.49 (21.85)	16.26 (15.63)
Women	3.11 (3.07)	4.66 (5.43)	21.88 (22.13)	17.05 (16.58)
Age				
18–22	3.57 (3.45)	4.61 (5.43)	28.26 (23.65)	21.14 (18.26)
23–29	3.54 (3.19)	5.02 (5.76)	25.90 (22.70)	19.48 (17.77)
30–39	2.76 (2.78)	4.48 (5.46)	20.04 (21.06)	15.20 (14.09)
40–49	2.86 (2.92)	4.48 (5.31)	15.21 (18.28)	13.10 (13.30)
50–59	3.14 (3.03)	4.68 (5.22)	13.41 (18.44)	12.80 (13.67)
60+	3.49 (3.37)	5.80 (6.31)	10.91 (18.04)	12.88 (14.42)
Self-esteem				
Low	2.94 (3.17)	4.31 (5.50)	27.61 (25.40)	21.21 (19.57)
High	3.39 (3.19)	4.89 (5.54)	18.74 (20.52)	15.20 (14.80)
Self-control				
Low	3.19 (3.51)	3.91 (5.09)	31.13 (27.38)	22.77 (21.11)
High	3.22 (3.14)	4.96 (5.71)	18.33 (20.80)	15.09 (15.41)
Emotional stability				
Low	3.03 (3.21)	4.21 (5.21)	26.32 (24.30)	19.3 (17.93)
High	3.44 (3.22)	5.18 (5.87)	17.06 (19.53)	14.78 (14.27)

**Table 3:** Bivariate correlations between psychological factors, leisure activities, and satisfaction.

	**1**	**2**	**3**	**4**	**5**	**6**	**7**	**8**	**9**	**10**	**11**	**12**	**13**
Psychological factors													
1. Self-esteem	1												
2. Self-control	0.28 ***	1											
3. Emotional stability	0.48 ***	0.43 ***	1										
Leisure activities													
4. Sports	0.08 ***	0.00	0.04 ***	1									
5. Hobbies	0.05 ***	0.03 **	0.06 ***	0.20 ***	1								
6. Series/videos	−0.16 ***	−0.12 ***	−0.12 ***	0.02 *	0.03 **	1							
7. Social media	−0.16 ***	−0.14 ***	−0.16 ***	0.00	−0.05 ***	0.42 ***	1						
Satisfaction													
8. Family	0.38 ***	0.12 ***	0.25 ***	0.06 ***	0.03 **	−0.11 ***	−0.10 ***	1					
9. Friends	0.33 ***	0.07 ***	0.21 ***	0.10 ***	0.05 ***	−0.05 ***	−0.05	0.47 ***	1				
10. Work	0.44 ***	0.11 ***	0.22 ***	0.10 ***	0.03 **	−0.10 ***	−0.08 ***	0.34 ***	0.36 ***	1			
11. Leisure	0.34 ***	0.10 ***	0.23 ***	0.20 ***	0.16 ***	−0.02	−0.06 ***	0.28 ***	0.32 ***	0.41 ***	1		
Control variables													
12. Sex	−0.06 ***	−0.03 **	−0.15 ***	−0.06 ***	−0.02	0.02 *	0.05 ***	0.03 **	0.05 ***	0.00	−0.04 **	1	
13. Age	0.28 ***	0.21 ***	0.24 ***	−0.04 ***	0.03 **	−0.20 ***	−0.28 ***	0.17 ***	0.06 ***	0.14 ***	0.14 ***	−0.01	1

* *p* < 0.05; ** *p* < 0.01; *** *p* < 0.001.

**Table 4:** Total effects of psychological factors on leisure activities.

	Sports	Hobbies	Social Media	Series/Videos
	*β ^a^*	*β ^a^*	*β ^a^*	*β ^a^*
Self-esteem	0.09 ***	0.03 **	−0.05 ***	−0.09 ***
Self-control	−0.02 *	0.00	−0.05 ***	−0.05 ***
Emotional stability	0.02	0.04 ***	−0.06 **	−0.01
Sex	−0.05 ***	−0.01	0.04 ***	0.01
Age	−0.07 ***	0.01	−0.24 ***	−0.16 ***
Stringency	0.00	−0.03	0.02	0.03

*^a^* Standardized coefficient; * *p* < 0.05; ** *p* < 0.01; *** *p* < 0.001.

**Table 5:** Total and indirect effects on satisfaction.

	Family *β ^a^*	Friends *β ^a^*	Work *β ^a^*	Leisure *β ^a^*
**Total**				
Self-esteem	0.323 ***	0.300 ***	0.425 ***	0.288 ***
Self-control	0.002	−0.001	0.000	−0.005 **
Emotional stability	0.085 ***	0.087 ***	0.016	0.082 ***
Sports	0.041 ***	0.073 ***	0.066 ***	0.154 ***
Hobbies	0.004	0.015	−0.003	0.103 ***
Social media	−0.011	0.002	0.009	−0.002
Series/videos	−0.043 ***	−0.007	−0.031 *	0.038
Age	0.054 ***	−0.045 **	0.013	0.039
Sex	0.062 ***	0.078 ***	0.020	−0.007
Stringency	−0.028	−0.068 *	−0.015	−0.015
**Indirect**				
Self-esteem	0.008 ***	0.008 ***	0.008 ***	0.014 ***
Self-control	0.002	−0.001	0.000	−0.005 **
Emotional stability	0.002	0.002	0.001	0.007 ***
Age	0.007	−0.004	−0.002	−0.016 *
Sex	−0.003 **	−0.004 **	−0.003	−0.008 **
Stringency	−0.002	−0.001	−0.001	−0.002

*^a^* Standardized coefficient; * *p* < 0.05; ** *p* < 0.01; *** *p* < 0.001.

## Data Availability

The data presented in this study are available on request from the corresponding author. The data are not publicly available due to ongoing analyses.
